# Reducing patients’ rate of frequent attendance through a training intervention for physicians

**DOI:** 10.1186/s12909-024-05748-w

**Published:** 2024-07-14

**Authors:** Alex Ramos, Ramon Pujol, Carol Palma

**Affiliations:** 1 Postgraduate and Continuing Education in Health Sciences, Faculty of Life Sciences,, Universidad Nebrija, Madrid, Spain; 2 Continuing Medical Education Centre of the Official Medical College of Barcelona, Barcelona, Spain; 3 Internal Medicine, Faculty of Medicine of the University of Vic-Central Catalonia, Barcelona, Spain; 4 Board of Directors of the Official Medical College of Barcelona, Barcelona, Spain; 5 Psychology at Blanquerna Faculty, Universitat Ramon Llull, Barcelona, Spain; 6 Psychologist at the Mental Health Center of the Mataró Hospital, Barcelona, Spain

**Keywords:** Frequent attenders in primary care, Evaluation of an educational intervention, Effectiveness of continuing medical education (CME), Impact, outcomes in physicians and in patients

## Abstract

**Background:**

Frequent attendance is a common issue for primary care health centres. The phenomenon affects the quality of care, increases doctors’ workloads and can lead to burnout.This study presents the results of an educational intervention for primary care physicians, aimed at helping them to decrease the prevalence rate of excessive attendance by patients at their centres.

**Methods:**

A training programme was carried out for 11 primary care doctors in Barcelona who had patient lists totalling 20,064 patients. The goal of the training was to provide the participating physicians with techniques to curb frequent attendance. Additionally, the programme sought to offer them strategies to prevent professional burnout and tools to better organize their everyday medical practice. The study used a quasi-experimental design for an evaluation of an educational intervention, featuring a pre-test assessment (before the training programme) and a post-test assessment (after the training programme), as well as comparison with a control group that did not undergo the training. The study assessed the effects of the programme on the rates of frequent attendance of patients served by the participating physicians. These rates were compared with those registered by the patients seen by the control group physicians over the same period.

**Results:**

Among the group of physicians who received the training, the mean prevalence of patients who qualified as frequent attenders decreased from 22% prior to the training programme to 8% after completion of the programme. In other words, 14% of patients (2,809) limited the frequency of their visits to primary care physicians after their physicians had completed the training programme. Meanwhile, the study recorded an average decrease of 3.1 visits per year by the patients of the physicians who had undergone the training. Statistically significant differences between this group and the control group were observed.

**Conclusions:**

The educational intervention proved effective at helping primary care physicians to decrease their patients’ rates of frequent attendance. It also contributes to the impact research of continuing education on doctors and their patients. We need to increase primary care spending from the current 14% to the 25%, to address this problem, among others.

## Background

The demand for medical attention emerges from a person’s perception of discomfort or illness, and this is what drives the use of health services [1–13]. Frequency of attendance is an indicator measuring the mean number of visits that patients make to a physician’s office over a given period. The overall mean frequency of attendance in Spain has been calculated at slightly over five visits a year per patient, according to Bellón et al. [14, 15].

There is no single consensus definition of frequent primary care attendance, but some of the definitions offered by previous research include:

- Making over a certain number of visits per year. Generally, researchers set the threshold at 5–12 visits [5, 8, 9, 14, 16–19].
- Number of annual visits above a certain percentile within a patient list [2, 3, 8, 10, 20].
- Visiting a doctor’s office at least twice as often as the average patient [15, 21].

Other studies have defined frequent attendance not only in terms of the number of visits to a centre but also as a function of whether these visits are unjustified [9, 16, 17]. According to Gili et al. [22], 15–25% of primary care visits are made by frequent attenders. Elsewhere, a study by Smits et al. [2] found that primary care doctors tend to devote 80% of their time to just 20% of their patients.

The following factors have been found to be linked to frequent attendance:

- *Patient-based factors*. With an habitual pattern of chronic illness, psychological distress or social vulnerabilities [2–4, 6, 11–14, 17–20, 22, 23].
- *Organizational factors*. Related with the accessibility to the health centre, the ratio of physicians to inhabitants, the services offered and continuity of care [1, 14, 24–26].
- *Physician-based factors*. These have received little attention from researchers. Some studies have explored links between frequent attendance and individual physicians’ characteristics: patient-oriented or disease-oriented, age, gender, education, experience, personality, job security, decision-making skills and differences in how they organize their work, the ability to listen and the complacency to prescribe drugs [18, 24, 25].

On the other hand, the long period of continuing medical education is not a formalized mode of education. It is rooted in theories of Adult Education (Andragogy) [27, 28]. In this context, professionals themselves decide whether, how, where, how much and when to study. This is distinct from initial and formal educational practices (Pedagogy) aimed at child and younger learners, because in continuing education the focus is more on learning than on teaching.

Continuing education has adopted the guiding principles of adult education [29–35]: students are active participants and contribute to their own learning goals; learning is connected to professional needs; continuous training must have an immediate practical application; students must receive constant feedback from teachers; and those responsible for CME must practice effective adult education.

Several studies have sought to assess the effectiveness of different training activities. In this context, the evaluation is defined as the process whereby it is possible to determine the value or utility of a certain practice [36, 37]. In the medical field, there is a well-established body of scientific research assessing the effectiveness of continuing education [30, 31, 33, 39–53]. Also, in the fields of Psychology and Education [38].

Research by Norcini et al. [54] and Bordage et al. [55] has linked effective professional performance to knowledge acquired in training programmes. Bordage et al. [55] have stressed that to ensure that the effects of knowledge acquired in the context of CME are able to shape real clinical practice, it is important for this training to go beyond mere theoretical content. In other words, training programmes should strive to embrace applied knowledge, as this is more likely to lead to deeper learning and contribute to expanded professional expertise.

Davis et al. [31] found widespread consensus that CME has the potential to improve physicians’ professional performance, although the literature shows that training is more effective at helping doctors acquire new knowledge than performance.

The greatest difficulty when it comes to assessing CME lies in demonstrating how training interventions aimed at physicians actually affect patient outcomes. Certain studies, such as those by Marinopoulus et al. [44], Mazmanian et al. [45] and Davis et al. [48], have looked at how CME affects patients, but these researchers acknowledge the difficulties inherent in establishing whether a specific variable is definitive in shaping clinical outcomes, as a wide range of factors tend to come into play.

This study arose from a problem observed by the staff of a primary care centre in Barcelona, who wished to address the excess of visits from certain frequently attending patients through a specific training programme for its physicians. In response, this study was undertaken with the main objective of carrying out a training programme for physicians and subsequently evaluate the intervention impact on their patients. A comparison was also conducted between these patients and those of a control group of physicians who did not take part in the training.

In addition to this broader objective, the study sought to analyse the previous needs of the population of physicians that was to receive the training and to collect data on how the participating physicians assessed the training intervention.

## Methods

### Design

The study was designed as a quasi-experimental evaluation of an educational intervention, featuring a control group, and including pre–post assessments of both the intervention group and the control group.

### Setting

Between 2006 and 2018, the Centre of Studies of the Official College of Physicians of Barcelona, as a CME department under the direction of the first investigator of this study, included in its regular catalogue of training courses a programme entitled “Frequent attendance of primary care: Current status, approach and prevention”. This Centre had received a request from the administrators of the three primary care centres (CAP Valencia, CAP Monumental and CAP Ausias March) included in the Health Area of Dreta del Eixample of Barcelona in 2006, to organize a training intervention to help their medical staff manage the frequent attendance.

### Participants

The primary care physicians of these centres serve a total population of 51,934 patients. The total sample of patients studied was 20,064 (38.6% of the users), representing the patient lists of the 11 physicians who took part in the intervention. They had an average patient list of 1,824 patients per doctor.

The potential sample of participating physicians was 20, after 13 doctors had been excluded because they did not have a patient list or were specialists. Of the 20 eligible physicians, 11 (55%) agreed to complete the training programme.

### Recruitment

An objective account of a group’s existing needs is a necessary precondition for an effective educational intervention. With this in mind, a descriptive analysis was conducted in the three primary care centres. The objective was to gain a fuller picture of the profiles of the professionals who would be the recipients of the training, as well as of the centres where they worked and the patient population.

For the purposes of analysis, the threshold for overuse was set at more than 10 visits a year by a patient with his or her primary care physician, in accordance with determinations most frequently applied in the literature.

Based on the need’s detection process described above, it was decided that it was not feasible for us to conduct interventions aimed at the organization of the centres or directly to patients. Therefore, it was determined that a training intervention aimed at physicians would be the most viable, agile, and workable way to address the problem.

### Study intervention

The educational intervention was designed and implemented in a series of three distinct time periods.

1. Pre-training period

- Definition and selection of individuals responsible for research and training; design of the intervention; organization and logistics; creation of teaching materials; securing spaces; assignment of administrative support tasks; scheduling; selection process of teachers with experience in the field; experience in training and mastery of communication tools. The resulting teaching team was made up of two physicians, included the director and three psychologists, all of whom contributed to shaping the objectives, content and teaching methodology of the programme.

2. Intervention period:

- *Teaching objectives*: become familiar with the phenomenon of frequent attendance in primary care; raise awareness of the magnitude of the problem; learn techniques to manage frequent attendance (how to reduce the number of visits); and learn instruments and strategies to prevent job burnout.
- *Training content*: (a) information on overuse based on a literature review, available data, limits and gaps; (b) strategies for dealing with patients who overuse resources and professional skills to improve communication with patients (active listening, coaching and counselling); (c) strategies to prevent job burnout, including assertiveness, time management and self-knowledge; and (d) organization and management of one’s own medical practice.
- *Teaching methodology*: a combination of lectures and practical interactive workshops; a total of 16 h split into two immersive training sessions of 8 h each, held in the classrooms at the CME Centre in July and October 2006. The course was offered free of charge, and physicians were able to attend during their regular working hours.

3. Post-training period:

-The participating physicians assessed the training programme and a study of the effects of the educational intervention on their patients was carried out.

### Outcome measures

The main outcome measures were: the number of visits made by each patient over the period of the study (one year); and the percentage of patients who attended more than 10 times a year.

Additionally, measures of training expectations of physicians were collected with a Pre-training Questionnaire and satisfaction measures with the Post-training Questionnaire.

### Data collection

To make possible the identification of needs and analysis of the effects of the training programme, the participants were asked at the start and at the end of the process to complete the Pre-training and Post-training questionnaire respectively.

The study compared the pre- and post-test data of the patients attended by the doctors in the intervention group. Data was in an anonymized form. These data were also compared with those of a control group, made up of patients from another primary care centre (CAP Passeig Sant Joan) in the same neighborhood in Barcelona (*Eixample*) whose physicians had not undergone the intervention.

Trying to avoid data collection bias, the physicians who took part in the training and the teachers they didn’t know the study of the impact in patient outcomes.

For the evaluation of the impact of the educational intervention, data provided by the administrators and IT specialists of the primary health centres were used. Data on the following variables were collected: number of patients assigned to each physician (list) and physicians’ age, gender, specialization in family medicine or not and working hours.

### Instruments/data sources

Two ad hoc questionnaires (created for the purposes of this study) were used to allow the physicians to assess the training intervention: (1) Pre-Training Questionnaire ; and (2) Post-Training Questionnaire. The Pre-Training Questionnaire, asked for expectations via the item: “What do you hope to achieve in this training programme?”.

Likewise, Post-Training Questionnaire, assessed the degree to which their satisfaction had been met by gathering feedback on the course’s methodology, training resources, and usefulness, as well as the extent to which the participants thought what they had learned would be applicable in practice. This questionnaire consisted of 10 items, specified in Table 1 each of which was answered on a five-point Likert scale.

### Data analysis

For detection and analysis of the training needs of the target population, a Pearson bivariate correlation analysis was carried out to investigate the associations between measurements of physician activity and the rates of frequent attenders (dependent variable). A univariate analysis was also carried out to identify any factors that might influence or predict frequent attendance. A series of confounding variables (population, patient lists and number of medical records in the centres) were introduced into the calculations to correct for any potential bias.

For the study of the impact of the training intervention on the frequent attendance patterns of the physicians’ patients, statistical analysis made it possible to compare the frequent attendance indicators identified above in the intervention and control groups in Periods 1 and 2. Additionally, covariate analysis was conducted to take into account any differences in terms of the characteristics of the doctors or their patient lists: gender, age, specialization, working hours and patient list size (mean).

Data were analyzed via the calculation of Pearson correlations, non-parametric intergroup and intragroup comparison tests, and analysis of variance (ANOVA), to observe the interaction effects of covariables on the differences between groups.

The qualitative data from the Pre-training Questionnaire consisted of the grouping of responses around the meaning “The doctor participating in the training is concerned about the frequent attendance of patients and wants to acquire tools to manage it.” The qualitative data of the Post-training Questionnaire were obtained from the response means around the 10 items with a scale of 1 to 5 in each response.

### Ethical considerations

It did not use personal data of patients. The data used for the study came from the record of aggregated data on the frequent attendance in common use by the participating centres. Data was provided in an anonymized form.

## Results

### Baseline characteristics of TIG and CG groups

No statistically significant association was found between the gender of the participating physicians and the degree of frequent attendance of their patients (Kruskall-Wallis *H* = 0.778; *p* = 0.378). Similarly, no statistically significant links were found between the professionals’ age and the extent to which their patients overuse services, although it is true that in this case the data point to an inverse tendency.

(*r* = − 0.32; *p* = 0.147).  Younger physicians tend to experience more frequent attendance than their older colleagues. Finally, no statistically significant differences were found either as a function of the specialization of family physician (Kruskall-Wallis *H* = 0.564; *p* = 0.754) or as a function of their working timetables (Kruskall-Wallis *H* = 2.035; *p* = 0.154).

While they do not have statistically association value, the patient variables associated with higher levels of frequent attendance were as follows: making unscheduled doctor’s visits (*r* = 0.920; *p* = 0.000); making appointments with the centre’s receptionist (*r* = 0.853; *p* = 0.000); being over 64 years of age (*r* = 0.663; *p* = 0.000); having higher levels of healthcare costs (*r* = 0.473; *p* = 0.011); and having pharmacological prescriptions (*r* = 0.540; *p* = 0.003).

It is also worth noting that there was a stronger association between the presence of frequent attendance and the making of unscheduled visits (*r* = 0.920; *p* = 0.000) than there was with visits made with prior appointments (*r* = 0.527; *p* = 0.004).

The variables that were predicted by more frequent attendance were patient healthcare costs (*F* = 5.924; *p* = 0.025) and the size of patient lists (*F* = 12.882; *p* = 0.037). The variables that did not display any associations with overuse of patients in the correlation analysis or in the univariate analysis were telephone consultations (*F* = 0.421; *p* = 0.545), scheduled visits (*F* = 3.143; *p* = 0.174), waiting times (*F* = 0.002; *p* = 0.967) and consultation times (*F* = 0.2; *p* = 0.685).

Finally, the analysis showed that 18.7% of patients account for over 50% of visits and consultations.

### Pre-and post training results

For the open-ended item on the Pre-Training Questionnaire, all of the 11 participating physicians provided spontaneous answers along the same meaning, indicating that “Frequent attendance is a concern and that they would like to have tools to manage it”. Specifically, they expressed an interest in a training programme that would allow them to acquire practical tools; a programme that would meet their need for an immediately applicable approach to the issue; teach them techniques and strategies to reduce overuse; help them control job stress; improve their relationships with patients; provide them with strategies for early detection of patients with the potential to overuse resources and help them to improve the quality of their doctor–patient interviews.

All the participants completed the Post-Training Questionnaire upon completion of the course and the mean responses are displayed in Table 1. The data show a high degree of overall satisfaction (mean response of 4.8 out of 5) with the course, and high rates of approval of the teachers and training approaches (organization, teaching staff, interaction, and satisfaction with sessions). After the course a mean response of 4.2 out of 5 of participants reported that they were motivated to apply what they had learned to their professional practice to manage frequent attendance.

**Table 1:** Mean responses to the Post-training Questionnaire

Items measuring satisfaction	Mean*
Prior expectations met	4.0
Applicability of learning/knowledge to professional practice	4.2
Impact of training on professional performance	4.4
Motivation to expand on training received	4.0
Organization of course	4.8
Teaching staff	4.9
Teaching materials	3.8
Student–teacher interaction	4.9
Satisfaction with sessions and case studies	4.1
Overall satisfaction	4.8

*On a Likert scale from 1 to 5

### Pre and post for training intervention group vs. control group

An analysis was carried out of the baseline characteristics of the two groups of physicians, namely, the training intervention group (TIG) and the control group (CG) (see Table 2).

**Table 2:** Baseline characteristics of the training intervention group (TIG) and *control group (CG)*

Group	Gender (%)		MIR (%)		Working hours	Age	Patient list
	Females	Males	Yes	No	Hours/day	Mean (SD)	Mean (SD)
**TIG** (*N* = 11)	64	36	91	9	7	39 (9.24)	1824 (119.96)
**GC** (*N* = 13)	23	77	69	31	7	50 (10.4)	1747 (275.76)

MIR, Resident Internal Physician/Specialization; SD, standard deviation

The baseline data (covering Period 1, the 12 months prior to the training course) were analyzed to investigate any differences between the groups in terms of patient list size, mean visits per patient prior to the training and proportion of frequently attending patients. Regarding patient list size, no significant differences were found between the TIG and CG (Mann-Whitney *U* = 68.00; *p* = 0.865). However, the baseline data did indicate differences between the groups in terms of number of visits per patient, with the TIG showing a higher mean number of visits per patient and a greater proportion of frequent attenders (see Table 3).

The TIG’s results for Period 2 (covering the 12 months after the intervention) show statistically significant reductions with respect to the baseline scores in the measurements of mean visits per patient and mean proportion of frequently attending patients. Meanwhile, in the CG, these measurements (mean visits per patient and mean proportion of frequent attenders) remained unchanged over the two periods (see Table 3 ).

**Table 3:** Comparison of the mean annual number of visits per patient and the mean proportion of frequently attending patients in periods 1 and 2, in the training intervention group (TIG) and control group (CG)^3^

Group	Mean visits per patient (SD) Period 1	Mean visits per patient (SD) Period 2	Z	*p*	Mean proportion of frequent attender patients (SD) Period 1	Mean proportion of frequent attender patients (SD) Period 2	Z	*p*
TIG (*N* = 11)	6.69 (0.79)^1^	3.59 (0.86)	-2.93	0.003*	0.22 (0.07)^2^	0.08 (0.04)	-2.9	0.003*
CG (*N* = 13)	4.68 (1.31)^1^	4.58 (0.46)	-0.39	0.695	0.06 (0.19)^2^	0.05 (0.02)	-0.664	0.507

*Statistically significant at 0.01 using the Wilcoxon test. SD, standard deviation

^1^*p* < 0.0005

^2^*p* < 0.0005

^3^ Note: The mean visits per patient was significantly lower in the TIG group compared to the CG group in period 2 (U = 23; *p* < 0.0005). No differences were detected in the mean proportion of frequent attender patients between the groups (U = 48; *p* = 0.186)

The mean proportion of frequently attending patients in the TIG was 0.22 (4,414 patients) in Period 1 but this figure dropped to 0.08 in Period 2 (1,605 patients). In absolute terms, this reduction means that 14% of these physicians’ patients (2,809) regulated the frequency with which they were attended by their primary care doctors in Period 2, after these doctors had completed the training (see Table 3 ).

Comparison of the annual mean number of visits per patient in the TIG and CG in Period 2 indicates a decrease of 3.1 visits per patient in the TIG. This represents a reduction of nearly half, as the figure declined from 6.69 visits per patient in Period 1 to just 3.59 in Period 2. This decrease reflected a statistically significant difference compared to the CG .

In the CG, in fact, no differences were found in the mean proportion of frequently attending patients at the end of Period 2, meaning that the differences observed in this respect in the baseline measurements of the groups were no longer significant in Period 2.

The two groups both had a similar mean proportion of frequent attenders at the end of Period 2, with the TIG displaying a clear decrease with regard to its baseline score. Meanwhile, the two groups displayed statistically significant differences in Period 2 in the annual mean number of visits per patient, with the TIG recording the lower figure.

These statistics are evidence that the training programme was able to achieve its objective of reducing the rate of attendance at the intervened primary care centres in the area.

The analysis shows a link between completion of the training programme and the decrease in the frequency of visits. Among the patients who reduced their number of visits are the so-called frequent attenders, as defined by the established criteria applied here.

## Discussion

This study has demonstrated the effectiveness of an educational intervention for primary care physicians as a tool to reduce frequent attendance by patients.

The article details the real-life experience of an Adult Education course, in this case aimed at the physicians from three primary care centres in Barcelona. The doctors who enrolled in the course were driven by a pressing issue emerging from their everyday practice, the need to manage the frequent attendance at their offices.

The main objective, then, was to address a specific request from part of this public health service to reduce the number of visits made by frequent attenders, a concern that is also widespread in the literature [2, 5–7, 10, 17, 19, 20, 22].

The study also wanted to contribute to the field of research on the evaluation of educational interventions on physicians and their patients [30, 31, 39–46, 48–50, 52, 53].

While 11 physicians completed the training course, the effects and impact of the intervention were felt in a large sample of 20,064 patients, representing 38.6% of the total population of patients of these centres.

The study employs a quasi-experimental design with pre- and post-evaluation and a control group. This approach is among those most often used to assess public health programmes and training interventions in contexts where the random assignment of individuals to groups is not possible [50, 57–59]. According to León et al. [38], while it is true that quasi-experimental studies have a lesser degree of internal validity than experimental studies because they exert lesser control over variables, the former have greater external validity because they are carried out in real-world settings. A two-phase reasoning process [57, 58] should be used to help in consider the effectiveness of the intervention with this design: (1) *suitability of the intervention* – the unlikelihood of any alternative explanations observed after the intervention, supported by: the magnitude of the change is very clear, the causal chain between the intervention and its effects is direct and these effects occur just after the intervention ; and (2) *plausibility* – compare with a control group that is similar to the intervention group but differs in terms of the independent variable (with or without the training intervention).

The baseline comparison between the intervention group and the control group showed that the two were homogeneous in terms of the following variables: specialization profile, work timetable (full- or part-time status) and size of patient list. There were differences between the groups in terms of the gender and age of the physicians, but it should be noted that the results in Phase 1 indicated no statistically significant relationship between doctors’ gender and the rate of frequent attendance of their patients. There was, however, a slight (but not statistically significant) tendency for younger doctors to experience more patient frequent attender.

One of the important implications of this study is that a small percentage of frequent attenders (18.7% of all patients) account for over 50% of all healthcare activity. This helps to explain the demand of the primary care centres administrators. This finding echo those of other studies that have highlighted the high cost of frequent attendance in the public health system, contributing to an already large workload, and affecting physicians and ultimately their patients [2, 9, 10, 12, 20, 22]. Cebrià et al. [60] have linked the professional burnout by frequent attendance with a greater likelihood of medical errors that can increase in pharmaceutical expenses.

This research focuses on the potential of educational intervention to address this problem and evaluates the impact of training on physicians and their patients [31, 35, 40, 43–45, 48], specifically in the context of primary care [52, 53]. Physicians’ training has been related with the impact in how they manage frequent attendance of their patients [5, 7, 17, 61].

The training programme outlined here puts special emphasis on improving doctor–patient communication and the management of the doctor’s clinical office.

When asked about their expectations, the participating physicians observed that reducing frequent attendance by patients might be a preventive factor against job burnout, coinciding with the literature in the field [5, 10, 20, 62].

In the post-training questionnaire, the physicians reflect their motivation. In cases of frequent attendance, the physician is often aware of what is happening but does not know how to respond, and they hoped that this customized course would help them to address this problem. The motivation serves to drive the doctors to act, but it is not enough, and the training programme channels them toward a solution.

Also worth highlighting is that training is often more successful when is designed in collaboration with the students and based on detection of the needs from their everyday practice. In this way, the learning acquired can be applied directly in the workplace to meet existing needs. This training approach is based on the principles of adult education [28, 31–34, 40, 41]. Bennet [40] observes that when a person is aware of his or her training needs, it is as easy as starting the training process.

According to Arnold-Rehring [63], there is a greater likelihood that training will lead to solve a problem and to meet needs if the professional him- or herself takes on a commitment to do it. Behavioral change can occur even over a very short period if the physicians themselves are committed to it.

The intervention in this study had satisfactory effects on the participating physicians as we observed in the post-training questionnaire with high rates of satisfaction and also with motivation to apply what they had learned into their professional practice.

Specifically, after the training period, the physicians who completed the intervention recorded both a significant decrease in the annual mean frequency of visits per patient (3.1 visits) and a 14% drop in the proportion of visits attributable to frequent attenders. These reductions are statistically significant both in terms of longitudinal analyses of the intervention group and in comparison, with the control group.

Among the limitations of this study was the fact that it was not possible to use an experimental design featuring the random assignment of participants, a design that would have allowed for the control of confounding variables. Hence, the results cannot be generalized beyond the sample analyzed in this study, but the findings here can be used to make estimates and guide future researchers in this field.

Being a quasi-experimental study, the motivation as a variable of the doctors is not controlled; we take this into account as a limitation of the study, although we consider it as a fertilized land factor that will facilitate a training intervention to achieve the desired result. We also consider age differences that may be associated with this variable,

Systematic reviews [8, 12, 13] on frequent attendance have drawn a clear situation of this phenomenon. A typical profile is described with chronic illness, advanced age, mental health, social vulnerabilities and pediatric attention with low family incomes. It is also recommended [8] to set the frequent attendance parameter starting at 10 annual visits. And the wide variety of results in this field makes comparisons difficult [9], and it is recommended to standardize the data on frequent attendance to allow for more reliable and rigorous comparisons between regions and countries [6].

The effectiveness of training and educational intervention on physicians and their patients, as described here, is comparable to what has been found in other recent studies using proven training methodologies [5, 49, 50, 53, 64, 65].

We want to highlight the focus of training intervention aimed at doctors, not at the organization of the centre and even less at the patients, who, apart from not blaming them, we should take into account their needs, psychosocial problems [66, 67] and the context to be more operational and avoid errors in the relationship [68]. To resolve the causes of frequent attendance based on patients and the healthcare organization, a much broader approach and at another level would be required.

## Conclusions

This study has allowed us to conclude that the training intervention was successful in its aim of reducing the rates of frequent patient attendance at the three participating primary care centres and considering the acceptable design limitations described above. Moreover, the physicians who completed the training programme rated it as fully satisfactory, seeing significant decreases in the annual proportion of frequent attenders among their patients and the annual mean number of visits per patient.

The communication and the office management training given to the physicians helped them to educate their patients about the responsible use of health services. Such tools, then, enable doctors to more effectively manage their patients’ care.

Considering the above, the data presented here provide a way forward to continued research on the evaluation of the educational intervention on physicians and their patients. This will help to ensure that training programmes are able to meet the needs of everyday medical practice with a quality improvement outcome, as was the case in this study addressing the frequent attendance of patients in primary care.

Frequent attendance it is one of the consequences of the insufficient distribution of health spending in the saturated primary care of our national health system. For years [69, 70] there has been unanimous agreement in society and among experts on the pressing need to increase primary care spending from the current 14% to the necessary 25%, to address this problem, among others issues in primary care in Spain .

## Data Availability

The data used and/or analysed for the purposes of this study are available from the author upon reasonable request.

## Abbreviations

CME: Continuing medical education
MD: Doctor of medicine
MEd: Master of education
PhD: Doctor of philosophy
TIG: Training intervention group
CG: Control group
